# Personal space regulation is affected by unilateral temporal lesions beyond the amygdala

**DOI:** 10.1093/texcom/tgac031

**Published:** 2022-07-22

**Authors:** Audrey Dureux, Luca Zigiotto, Silvio Sarubbo, Clément Desoche, Alessandro Farnè, Nadia Bolognini, Fadila Hadj-Bouziane

**Affiliations:** Integrative Multisensory Perception Action & Cognition Team - ImpAct, INSERM U1028, CNRS UMR5292, Neuroscience Research Center (CRNL), 69500 Lyon, France; University UCBL Lyon 1, University of Lyon, 69622 Lyon, France; Department of Neurosurgery, Azienda Provinciale per i Servizi Sanitari (APSS), “Santa Chiara Hospital”, 38122 Trento, Italy; Department of Psychology, Azienda Provinciale per i Servizi Sanitari (APSS), “Santa Chiara Hospital”, 38122 Trento, Italy; Department of Neurosurgery, Azienda Provinciale per i Servizi Sanitari (APSS), “Santa Chiara Hospital”, 38122 Trento, Italy; University UCBL Lyon 1, University of Lyon, 69622 Lyon, France; Hospices Civils de Lyon, Neuro-Immersion & Mouvement et Handicap, 69677 Lyon, France; Integrative Multisensory Perception Action & Cognition Team - ImpAct, INSERM U1028, CNRS UMR5292, Neuroscience Research Center (CRNL), 69500 Lyon, France; University UCBL Lyon 1, University of Lyon, 69622 Lyon, France; Hospices Civils de Lyon, Neuro-Immersion & Mouvement et Handicap, 69677 Lyon, France; Center for Mind/Brain Sciences (CIMeC), University of Trento, Trento, Italy; Department of Psychology, University of Milano Bicocca, 20126 Milano, Italy; Laboratory of Neuropsychology, IRCCS Istituto Auxologico Italiano, 20122 Milano, Italy; Integrative Multisensory Perception Action & Cognition Team - ImpAct, INSERM U1028, CNRS UMR5292, Neuroscience Research Center (CRNL), 69500 Lyon, France; University UCBL Lyon 1, University of Lyon, 69622 Lyon, France

**Keywords:** amygdala, facial emotional expressions, interpersonal distance, stop distance, virtual reality

## Abstract

We constantly face situations involving interactions with others that require us to automatically adjust our physical distances to avoid discomfort or anxiety. A previous case study has demonstrated that the integrity of both amygdalae is essential to regulate interpersonal distances. Despite unilateral lesion to the amygdala, as to other sectors of the medial temporal cortex, are known to also affect social behavior, their role in the regulation of interpersonal distances has never been investigated. Here, we sought to fill this gap by testing three patients with unilateral temporal lesions following surgical resections, including one patient with a lesion mainly centered on the amygdala and two with lesions to adjacent medial temporal cortex, on two versions of the stop distance paradigm (i.e. in a virtual reality environment and in a real setting). Our results showed that all three patients set shorter interpersonal distances compared to neurotypical controls. In addition, compared to controls, none of the patients adjusted such physical distances depending on facial emotional expressions, despite they preserved ability to categorize them. Finally, patients' heart rate responses differed from controls when viewing approaching faces. Our findings bring compelling evidence that unilateral lesions within the medial temporal cortex, not necessarily restricted to the amygdala, are sufficient to alter interpersonal distance, thus shedding new light on the neural circuitry regulating distance in social interactions.

## Introduction

In social situations, we automatically and continuously adjust our physical distance with others. This physical distance, termed personal or interpersonal space, depends on the relationships between individuals and the social context where the interaction takes place, for example if we take part in a family meeting, in work environments or in a crowded train, elevator, or street surrounded by strangers (Hall et al. 1968; Hayduk 1983).

In the laboratory, interpersonal space is typically assessed with the “stop-distance paradigm.” Participants face an experimenter located about 3 m away and moving toward them (or vice versa). They have to stop the experimenter (or themselves) nearest to the distance they start feeling uncomfortable with the others’ proximity. Violation of this invisible boundary creates a sense of discomfort in individuals. In healthy participants, the interpersonal distance typically varies between 60 and 160 cm depending on the condition (Iachini et al. 2014, 2015, 2016; Ruggiero et al. 2017). Its extent also depends on the participant’s personality traits and is also influenced by the confederates’ attributes, such as its age and sex (Iachini et al. 2015, 2016; Ruggiero et al. 2019) or their perceived emotional states (Ruggiero et al. 2017; Cartaud et al. 2018) or moral status (Pellencin et al. 2018).

Previous studies revealed that the amygdala is a critical node in the regulation of physical distances between individuals. The most direct evidence of its implication comes from a case study of a patient (SM) with a rare genetic disease (Urbach-wiethe), that leads to selective, bilateral damage to the amygdala (Kennedy et al. 2009). Compared to controls, SM did not feel any discomfort with the proximity of others. Her interpersonal distance with the experimenter was 34 cm, half shorter compared to that of neurotypical control subjects. In the same study, the authors reported increased amygdala activation as measured with functional magnetic resonance imaging (fMRI), when control subjects were made to believe that another person was close, as compared to far from them. Based on these findings, Kennedy and collaborators suggested that the amygdala is crucial for those feelings of discomfort associated with close physical proximity that help maintaining socially appropriate interpersonal distance. This proposal may also be related to the alteration in patients with bilateral amygdala lesions of physiological and subjective responses to emotionally salient stimuli, particularly fear-related responses (Adolphs et al. 1999; Davis and Whalen 2001; Masaoka et al. 2003; Ledoux and Brown 2017). This suggests that the feeling of discomfort when one violates our personal space might be triggered by the stimulation of the autonomic system following amygdala activation. This is supported by a recent study demonstrating that amygdala stimulation in control subjects strongly modulated autonomic activity (i.e. large changes in heart rate—HR) (Inman et al. 2020). This mechanistic explanation is also coherent with the role of amygdala in social approach and avoidance (Emery et al. 2001; Capitanio et al. 2006; Kennedy et al. 2009) and more generally, in its contribution to the processing of emotionally and socially relevant information (Martin and Weisberg 2003; Adolphs 2010; Noack et al. 2015).

To date, available evidence suggests that, compared to bilateral amygdala lesions, patients with unilateral amygdala damage exhibit less marked deficits (Adolphs et al. 1995) and the degree of impairment seem to depend on the side of lesion (Adolphs et al. 2001; Fowler et al. 2006). Yet, to the best of our knowledge, the impact of a unilateral lesion involving the amygdala onto the regulation of interpersonal distance has never been tested. Beyond amygdala, other brain regions of the temporal lobe are thought to play a role in social cognition (Sarubbo et al. 2015, 2020; Vilasboas et al. 2017; Duffau 2021), yet their implication in the regulation of interpersonal distances remains unknown. Bilateral temporal pole (TP) lesions in monkeys, induce marked deficits in social interactions, notably the loss of emotional attachments to their infants and to other conspecifics (Bucher et al. 1970; Kling and Steklis 1976). A similar clinical picture was reported in a case study describing a patient who underwent right temporal lobectomy and lost all emotional attachments to his family members, although he was still able to recognize them (Lipson et al. 2003). Given that electrical stimulation of the human TP, like the amygdala, alters autonomic responses such as HR, respiratory rate, and blood pressure, it is possible that the lesion prevented this physiological response associated with social attachment (Gloor et al. 1982).

We therefore suggest that the regulation of interpersonal distance that governs our social interactions may not be strictly dependent on the integrity of both amygdalae, but might rather depend on an interconnected network involving different regions of the temporal cortex. Here, we sought to nvestigate whether and to what extent unilateral temporal lobe lesion may cause deficits onto the regulation of interpersonal distances during social interactions. We thus characterized behavioral and physiological signatures of three patients with unilateral medial–temporal lesions following surgical resections, in the regulation of distance in socioemotional contexts. We specifically tested one patient with a unilateral lesion, which was mostly confined to the left amygdala (Patient P1), and two other patients with unilateral lesions to medial temporal cortex, sparing amygdala tissue (Patients P2 and P3). We further aimed to extend our understanding in personal space regulation by investigating whether a unilateral amygdala lesion elicits similar deficit in personal space regulation in different emotional contexts, also exploring the impact of a lesion largely confined to the amygdala to that of lesions comprising nearby regions of the temporal lobe.

We thus tested these patients on the stop distance paradigm in a virtual reality (VR) environment (experiment 1) and in a real setting (experiment 2). In both experiments, participants were asked to stop virtual faces or real confederates moving toward them at the nearest distance they start feeling uncomfortable with the confederates’ proximity. In Exp 1, faces depicted 3 different emotions: happy, neutral, or angry and participants’ HR frequency was recorded to additionally evaluate the impact of unilateral medial–temporal lesions onto their physiological state.

## Materials and methods

### Participants

We recruited 3 patients with postsurgical unilateral temporo-mesial resections (see Table 1 for details). Patients underwent awake (P1 and P2) or asleep surgery (P3) for glioma or metastasis resection at the Department of Neurosurgery of the Azienda Sanitaria per i Servizi Sanitari (APSS, “S. Chiara Hospital”, Trento, Italy). For awake surgery, the cortical and subcortical mapping was performed with 60 Hz, 1 ms and amplitude ranging between 2 and 4 mA, setting the threshold when eliciting speech arrest at the level of the ventral premotor cortex (VPMC; Zacà et al. 2018), stopping the resection when functional responses were elicited at cortical and subcortical stimulation of the eloquent structures; intraoperative neuropsychological assessment had been customized for every patient, as already reported (Dallabona et al. 2017; Zigiotto et al. 2020; Sarubbo et al. 2021). For the asleep procedure, the resection stopped after the complete resection of the enhancing area as shown on the MRI. All the surgeries were assisted by neuronavigation (Steatlh Station 7, Medtronic, Minneapolis, MN, USA). Before taking part in the experiments, all three patients signed an informed consent, and the study was approved by the ethical committee of the Azienda Provinciale per i Servizi Sanitari (Rep. Int. 14854 del 12 September 2019, Verbale VI/2019). Every patient underwent a complete neuropsychological assessment within 1 week of the experiment, including tests for Language, Attention, Executive functions, Memory, Visuo-spatial cognition, Praxis and, importantly, Social cognition, as previously reported (Zigiotto et al. 2020). Interestingly, none of them showed cognitive deficit in any domain, with the exception of P3 who had a mild impairment in long-term verbal memory (Rey’s 15 words delayed recall score under cut-off). Moreover, the Frontal Behavioral Inventory (FBI) (Alberici et al. 2007), a psychological scale useful to detect behavior and personality changes especially in behavioral fronto-temporal dementia, was administered to every patient’s caregiver (i.e. wife) in order to identify changes and/or inappropriate behavior in patient’s social interactions. For P1 mild/occasional changes in “aspontaneity,” “disorganization,” and “loss of insight” were reported; for P2 mild/occasional changes were observed in “apathy,” “indifference,” “perseveration,” “irritability,” and “restlessness,” with a moderate change in “inflexibility”; finally, P3 showed mild/occasional changes in “aspontaneity,” “indifference,” “disorganization,” “inattention,” “loss of insight,” and a moderate change “hyperorality.” Importantly, no difference was reported for any patient in “inappropriateness” items, which assess the ability to keep and maintain social rules.

**Table 1:** Details concerning each patient and their lesion site (i.e. location and ratio in percent for each brain region impacted).

Patients	Sex	Age	Education (years)	Type of surgery	Hemisphere	Testing since surgery (months)	Brain regions impacted by the lesion					
							PHC (%)	HC (%)	AMY (%)	FUSI (%)	TP (%)	MT (%)
P1	M	53	13	Awake surgery for Low Grade Glioma	Left	60	11.35	5.69	64.43	-	32.7	0.31
P2	M	58	13	Awake surgery for Low Grade Glioma	Left	10	7.48	-	-	8.14	4.83	11.37
P3	M	61	8	Asleep surgery for Renal Metastasis	Right	1	22.43	3.59	0.96	9.46	-	13.57

Brain regions damaged by the lesion: values represent the ratio (in percent) of lesioned voxels for each patient. This ratio corresponds to the number of lesioned voxels divided by the total number of voxels within each ROIs of the AAL3 atlas (Rolls et al. 2020).

PHC = parahippocampal cortex; HC = hippocampus; AMY = amygdala; FUSI = fusiform gyrus; TP = Temporal Pole; MT = Middle Temporal Gyrus.

**Fig. 1:**
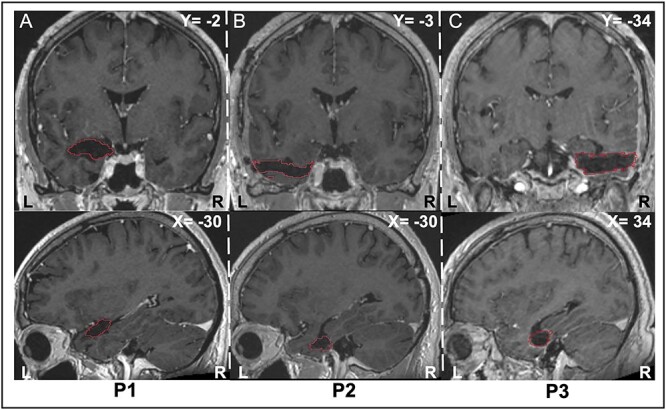
Postsurgical MR T1 images illustrating the surgical cavity of the unilateral medial–temporal lesions in the three patients. For each patient (A = P1, B = P2, C = P3), the lesions’ surgical cavities are outlined in red on anatomical T1 images in coronal (upper images) and sagittal (lower images) planes. (A) Patient P1 was submitted to a large resection involving the left amygdala extending toward the temporal pole, the middle temporal gyrus, the parahippocampal cortex, and the hippocampus. (B) Patient P2 was submitted to a resection centered to the left anterior temporal lobe including the temporal pole, the left middle temporal gyrus, the left fusiform gyrus, and the left parahippocampal cortex. (C) Patient P3 was submitted to a resection involving the right middle temporal gyrus, the right amygdala, the right fusiform gyrus, the right hippocampus, and the right parahippocampal cortex.

Concerning the lesion assessment, firstly, lesion surgical cavities were mapped by a neurosurgeon, blind to the experimental results, based on each subject structural T1 with gadolinium MRI, acquired within one month of the testing days. Then, lesion volume estimates were made by transforming each subject’ MR T1 to the standard MNI space using SPM (Friston et al. 2007) and were then aligned on the AAL3 atlas (Rolls et al. 2020). For each subdivision of the AAL3 atlas (i.e. 170 ROIs across the whole brain), we computed the percentage of lesioned voxels for each patient by dividing the number of voxels that intercepted between the lesion surgical cavity and each ROI by the total number of voxels within each ROI (see Table 1). Figure 1 shows the surgical lesions cavities superimposed on postsurgical MR T1 images of the three patients.

We compared the results of these 3 male patients to 10 neurotypical control males recruited through web advertising. They were selected to match patients on age and level of education age: 54.8 ± 4.98 (mean ± SD), education: 14.4 ± 1.65 (mean ± SD) and were tested on all the experimental tasks at the Impact laboratory (Lyon, France). All gave written informed consent and were paid for their participation. The study followed the Declaration of Helsinki standards and was approved by the Institut National de la Santé et de la Recherche Médicale (INSERM) Ethics Committee (IRB00003888, No. 16–341).

### Experimental protocols

To measure preferred interpersonal distances for each participant, we carried out 2 versions of the stop distance paradigm. One version was deployed in a virtual reality setting with approaching images of faces depicting 3 different emotions (experiment 1, virtual setting), after which we measured the participants” ability to categorize emotions depicted by the virtual faces (Validation task). The other version of the task involved a real setting in which the experimenter moves toward the participants (experiment 2, real setting). The aim of the second experiment was to assess the ecological validity of the VR experiment. Each participant performed these tasks following the same sequence: 1) Evaluation of preferred interpersonal distance in virtual setting (experiment 1, 25 min), 2) Emotional intensity rating (Validation task, 5 min), and 3) Evaluation of preferred interpersonal distance in real setting (experiment 2, 10 min).

### Stop distance paradigms: measure of interpersonal distance

#### Experiment 1: stop distance task in virtual reality setting

##### Apparatus and stimuli

Visual stimuli were presented in a VR environment. The scene was rendered in an Oculus Rift DK2 Head-Mounted Display (HMD), with a resolution of 960 x 1,080 per eye, a frequency of 75 Hz, a field of view equal to 106°. The HMD had an embedded 60 Hz eye-tracking system (SMI). The augmented technology Unity software (Version 5.1.2; Unity Technologies, San Francisco, CA) was used to create the virtual environment, display the stimuli and record participants’ responses. Visual stimuli were presented in the VR environment and participants provided responses using the index fingers of the dominant hand by pressing the spacebar on a computer keyboard. During the whole course of the experiment, a photoplethysmogram transducer (PPG, TSD200, Biopac) was attached to the right index finger of the participants to measure HR; the signal was recorded via a Biopac system (MP150, PPGED-EDA device) at a frequency of 1 kHz. Visual stimuli consisted of 4 individuals’ faces (2 females and 2 males) each mimicking 3 emotions (happy, neutral, angry). They were drawn from the Karolinska Directed Emotional Faces database (KDEF, Goeleven et al. 2008). We further processed these images in the following way: stimuli were cropped to an oval shape, to remove outline and external emotions (e.g. hairs); images were corrected for luminance, such that the mean luminance did not differ across sex or emotion of the faces. Each image measured 30 cm and the size was scaled as a function of distance as the faces were presented in looming direction.

##### Procedure

Participants sat in a quiet room, wearing a virtual reality headset (HDM). The VR environment consisted of an empty room, as to minimize the presence of distracting elements (Dureux et al. 2021). The instruction given to the participants was to: “press the button as soon as the distance between you and the face makes you feel uncomfortable.”

After a 5 points calibration of the eye-tracker, trials started with a white fixation cross, presented in the center of the visual field. If the subject correctly fixated the cross for 4,000 ms, its color became blue, and it was followed by the presentation of 1 face stimulus randomly chosen among male or female faces, its identity (2 levels for each sex) and its emotion (happy, neutral, angry). The virtual stimulus then loomed toward them at a constant speed (0.5 m/s) (Iachini et al. 2016; Ruggiero et al. 2017) until the participants stopped them by pressing the button and the face disappeared. The starting distance from the virtual face to the participant was randomized between 3 distances: 2.8, 3, and 3.2 m to avoid the habituation of the starting line for the participant.

Each virtual stimulus was presented a total of 36 times per emotion (18 for each male or female face), with a total of 108 trials, except for P3 where a total of 18 trials per emotion was presented (54 trials in total) to accommodate the patient’s condition and/or fatigue potentially induced by longer protocols. The task lasted about 25 min and a break could be introduced at any time during the experience by removing the HMD.

During the whole experiment, participants kept their left arm passively lying on the table in front of them with a photoplethysmogram transducer attached to their left index finger. The experiment started when the physiological signal (HR) appeared stable, typically from 5 s to 1 min, based on the experimenter’s visual inspection.

#### Experiment 2: stop distance task in real setting

##### Apparatus and confederates

The experimental session took place in an empty rectangular room in the Division of Neurosurgery, “S. Chiara Hospital” (Trento, Italy) for the testing of the three patients and in the Impact laboratory (CRNL, INSERM U1028, Lyon, France) for the testing of the healthy subjects.

For P1 and P2, one confederate, a female, took part in the study. For P3, in addition to the female confederate, a male confederate also took part in the study. For the healthy subjects, as for P3, the 2 confederates took part in the study. The confederates were unfamiliar to the patients and control group. During the experiment, the confederate wore neutral casual clothes without accessories. They were instructed to maintain a neutral expression, to keep their gaze on the chest of the participant and to move at roughly the same speed and arm swing while walking.

##### Procedure

The experiment was divided into 2 blocks, one active approach condition and one passive approach condition. In the active approach, the confederate remained motionless and participants walked toward them until they stopped. In the passive approach, participants stood still and saw the confederate walking toward them until they stopped the confederate.

In the passive condition, the instructions to the participants were similar to Exp. 1 (i.e. “stop the confederate as soon as the distance between you and the confederate makes you feel uncomfortable”). In the active condition, the instructions were to “stop as soon as the distance between you and the confederate makes you feel uncomfortable”.

The starting distance from participant to confederate was counterbalanced among 2.8, 3, and 3.2 m (as in the first experiment in VR). For P1 and P2, a total of 10 trials were performed with the female confederate only (5 trials in passive approach and 5 trials in active approach), as no male confederate was available for the day of testing for these patients. For P3 and healthy subjects, a total of 20 trials was performed, 10 trials with each male or female confederate (5 trials in passive approach and 5 trials in active approach). Once the participant stopped the confederate (passive-approach) or himself (active-approach), the chin-to-chin participant-confederate distance was measured using a digital laser distance measurer (Bosch GLM 20 Blaze 65′). At the end of each trial, the participant and confederate came back to the starting point. The order of blocks was counterbalanced across participants.

### Evaluation of emotional rating: validation task (VT)

We presented 18 stimuli sequentially, among which the 4 face stimuli that were used in experiment 1, on a 2D monitor screen and asked subjects to judge whether the displayed emotion was neutral, happy or angry using a 9 points Likert scale (range: −4/+4) presented below each face stimulus. In the scale, the minimum value (−4) indicated an angry face, the maximum (+4) a happy one, with intermediate values indicating neutral expressions. The open-source OpenSesame software (Mathôt et al. 2012) was used to display stimuli and collect responses. No time-limit was given.

### Data analysis

The variables of interests were analyzed with the open-source software R (The R Core Team 2013).

#### Interpersonal distance in VR settings

The mean distance (in meter) at which the participant stopped the virtual looming face was recorded in the different contexts of the task, varying 1) the facial emotional expressions (emotion: happy, neutral, and angry), 2) the Sex of faces (male or female), and 3) their Identity (ID 1 to ID 4). Patient P2 reported not feeling uncomfortable with the face stimuli looming toward him and never pressed the key to stop the face stimuli. In this case, negative values were obtained as the face crossed the boundary of the screen. The same happened in some trials with P3. We replaced negative values by 0 to facilitate graphical representation.

#### Interpersonal distance response in real settings

The mean participant-to-confederate distance (in meters) was recorded for the different conditions: 1) passive and active approach condition and 2) sex of the confederate (i.e. male or female).

#### Heart rate

We synchronized the HR signal with 2 triggers, one at the fixation cross onset at time 0 and one 4 s later, when the virtual faces appeared. We analyzed the HR signal with AcqKnowledge software (Biopac) to obtain average heartbeats per minute (bpm) for each participant in each trial (10 s). For that, we first detected all the inter-beat RR peaks automatically. Then, we manually inspected the signal and corrected for any mistakes with the automatic identification. The number of R peaks for 1 min corresponds to the number of contractions (beats) of the heart per minute (bpm), i.e. HR frequency. We computed the averaged heartbeats per minute (i.e. mean HR) during the entire period of stimuli presentation (i.e. onset: stimulus appearance; duration: 6 s interval with approaching faces) and also during the fixation cross period presented before the stimuli presentation in order to obtain baseline epochs (i.e. onset: fixation cross; duration: 4 s interval). Then, for each participant and for each trial, we computed the mean delta HR by subtracting each mean HR obtained during the period of stimulation by mean HR obtained in each baseline period.

The delta HR allows to determine the effect of face stimuli presentation on participants’ HR and to be able to compare these fluctuations between groups. As for the mean interpersonal distance, we also computed delta HR according to the different parameters of the task (i.e. emotion of faces presented, sex of faces, and identity of faces).

#### Emotional rating

The rating score (range: −4/+4) was analyzed for each facial emotional expression (emotion: happy, neutral, and angry) across the 4 facial identities.

### Statistical comparison between patients and controls

The variables of interest (interpersonal distances, delta HR, and emotional rating) of each patient were compared to the average score of the neurotypical control group using modified paired *t*-test specifically designed for single-case report, allowing to control for type I errors (Bayesian Test of Deficit) (McIntosh and Brooks 2011). The method described by Crawford and Garthwaite (2007) and Crawford et al. (2010) estimates, within a Bayesian framework, the point of abnormality of the patient’s score and the associated 95% credible limits (CL). In addition, this method provides the percentage of the healthy population obtaining a score lower than the patients. As recommended in Crawford et al. (2011), we chose to add covariates (i.e. level of education and age) when the correlation of a covariate with the dependent variable exceeded 0.3. Specifically, after assessing the distribution of our variables, non-parametric spearman tests were performed to compute these correlations between dependent variables and each with each covariate. For the VR experiment, we found that the level of education was negatively correlated with the mean interpersonal distances (*r* = −0.56, *P* = 0.093, BF = 1.1), while age was not (*r* = −0.10, *P* = 0.78, BF = 0.51) and we therefore included the level of education as a covariate. By contrast, for the experiment in real setting, neither the level of education nor the age correlated with the mean interpersonal distances (age: *r* = 0.28, *P* = 0.43, BF = 0.62; level of education: *r* = −0.22, *P* = 0.53, BF = 0.6) or with delta HR (age: *r* = −0.084, *P* = 0.83, BF = 0.51; level of education: *r* = −0.35, *P* = 0.36, BF = 0.53) and no covariate was included in these analysis. To perform Bayesian test of deficit with or without covariates, we then used the package *singcar* implemented on R studio with “BTD()” and “BTD_cov()” functions.

We also compared the patients’ and controls’ performances (i.e. interpersonal distance and delta HR) with mixed-effect multiple regression models using the lm4 package for R (Bates et al. 2015). We used linear mixed-effect models, a powerful tool in the analysis of single-case data to compare patients’ and controls’ scores (Huber et al. 2015; Wiley and Rapp 2019). As a first step, we defined a model containing the most appropriate random and fixed effects following the same procedure described in Blini et al. (2018) and Dureux et al. (2021). For the VR task, the final model included the group (i.e. patient and controls), the emotion type and their interaction as fixed factors and the subject, the sex of face stimuli and the starting distance as random factors. For the task in real settings, the final model included the group, the condition (i.e. passive or active) and their interaction as fixed factors and the subject and the experimenter (i.e. male or female) as random factors. Facial identity did not improve model accuracy and was therefore not included in the models. All post-hoc analyses for significant tests were computed on the basis of least-squares means (Lenth 2016), using False Discovery Rate (FDR, Benjamini and Hochberg 1995) correction for multiple comparisons. In all tests used (i.e. Bayesian test of deficit and linear mixed-effect models), a pre-specified significance level was set *α* = 0.05.

Finally, we performed Pearson’s correlations tests between mean interpersonal distance of the patient and control groups obtained in the two settings, real and VR, to assess their relationship.

## Results

### Behavioral data: evaluation of interpersonal distances with the stop distance tasks

**Fig. 2:**
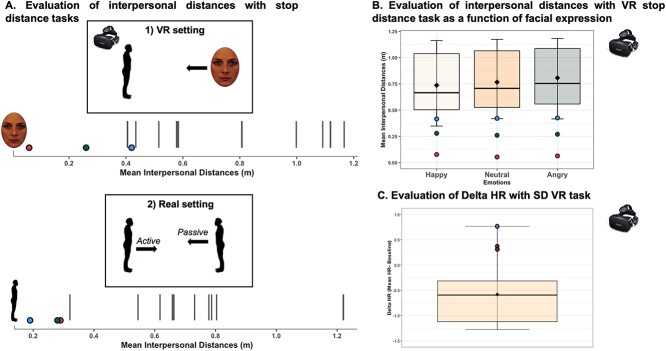
(A) Interpersonal distances evaluated with the stop distance paradigm in virtual reality (top panel, experiment 1) and real (bottom panel, experiment 2) settings in controls (*n* = 10) and patients (P1, blue dot, P2, red dot and P3 green dot). In each plot, the mean interpersonal distance (in meters) from the virtual faces (1) or the experimenter (2) is represented with vertical black bars for each control subject and each patient. (B) Interpersonal distances as a function of the emotion depicted by the faces assessed with the VR stop distance paradigm for the controls (*n* = 10) and patients (experiment 1). Mean interpersonal response distances (in meters) in controls are represented as box-plots for happy, neutral, and angry facial expressions. The three superimposed points represent the mean interpersonal distance for each patient. (C) Delta HR frequency in the VR stop distance paradigm (experiment 1) in controls (*n* = 9) and patients. Delta HR (i.e. mean HR during looming faces**—**mean HR during baseline period in bpm) in controls are represented as box-plots. The three superimposed points represent the delta HR for each patient. For (B and C), the vertical length of each box represents the interquartile range, the thick horizontal line represents the median, the whiskers indicate the full range of values and the black diamonds indicate the mean.

Figure 2A illustrates the preferred interpersonal distances of patients and controls in the VR and real setting experiments. Overall, all patients displayed a shorter mean interpersonal distance as compared to the control group in both settings—VR and real. Of note, there was a significant and strong correlation in mean interpersonal distance measured in the VR setting with looming face stimuli and in the real setting featuring a confederate (*r* = 0.76, BF = 14, *p* = 0.0026), confirming that performance in the virtual environment is well related to real-life situations.

The statistical comparison based of the Bayesian test of deficit (Crawford et al. 2011) of the mean interpersonal distances between each patient (P1 to P3) and the control group is presented in Table 2. In the VR setting, this analysis shows statistical differences between the control group and patients P2 and P3 in mean interpersonal distances (mean ± SD in m: controls = 0.77 m ± 0.30, P2 = 0.06, *p* = 0.01, zcc = −3.25 [95% CI: −4.77, −1.33], estimated percentage of control population falling below case’s scores = 1.14% [0.00%, 9.16%]; P3 = 0.27, *p*= 0.02, zcc = −4.28 [95% CI: −7.22, −0.78], estimated percentage of control population falling below case’s scores = 2% [0.00%, 21.78%]). P2 reported not feeling uncomfortable hence he did not press the key to stop the face stimuli moving forward, with mean interpersonal distance close to 0 m. A similar trend was observed for P1 with a shorter mean interpersonal distance compared to the control group, albeit this difference was not significant (P1 = 0.42, *p* = 0.07, zcc = −1.87 [95% CI: −2.94, −0.54], estimated percentage of control population falling below case’s scores = 7.27% [0.17%, 29.36%]).

**Table 2:** Comparison of interpersonal distances and mean delta HR (i.e. mean HR for looming faces—mean HR during fixation cross) between patients (P1, P2, P3) and controls (*n* = 10), assessed with the Bayesian test of deficit (Crawford and Garthwaite 2007).

Stop distance paradigms		Patients		Control group			One-tailed test
	Variables			*n*	Mean	SD	*p*
VR setting (Exp 1)^*^	Interpersonal distances (in m)	P1	0.42	10	0.77	0.30	0.07
		P2	0.06				^**^0.01
		P3	0.26				^*^0.02
	Delta HR	P1	0.77	9	−0.58	0.64	^*^0.04
		P2	0.37				0.09
		P3	0.33				0.11
Real setting (Exp 2)	Interpersonal distances (in m)	P1	0.19	10	0.71	0.23	^*^0.028
		P2	0.29				^*^0.05
		P3	0.28				^*^0.05

^*^We included the level of education as a covariate in the test as it correlated with interpersonal distances (*r* > 0.3). One-tailed test: *p*<0.05^*^, *p*<0.01^**^ and *p*<0.001^***^.

In the real setting, we also found statistical differences in mean interpersonal distances between the control group and the 3 patients [mean ± SD in m: controls = 0.71 ± 0.23, P1 = 0.19, *p*= 0.03, zcc = −2.29, 95% CI: −3.49, −1.07], estimated percentage of control population falling below case’s scores = 2.79% [0.02%, 14.16%]; P2 = 0.29, *p* = 0.05, zcc = −1.86 [95% CI: −2.87, −0.79], estimated percentage of control population falling below case’s scores = 5.44% [0.21%, 21.43%]; P3 = 0.28, *p* = 0.05, zcc = −1.87 [95% CI: −2.91, −0.77], estimated percentage of control population falling below case’s scores = 5.43% [0.18%, 21.99%].

To further compare the effect of conditions (facial emotional expressions in the VR setting and type of approach—active versus passive—in the real setting) in mean interpersonal distances between the patients and the control group, we employed mixed-effect GLMs.

In the VR settings, this analysis revealed a significant main effect of Group [*F*(1,11.02) = 7.89, *p* = 0.02] and Emotion [*F*(2, 1328.73) = 14.58, *P* < 0.001] and a significant interaction of Group*Emotion [*F*(2, 1328.73) = 4.04, *p* = 0.02]. As revealed with the Bayesian test of deficit analysis above, the control group preferred larger interpersonal distances compared to patients (controls–patients: *z* = 2.81, *p* = 0.005). Post-hoc test indicated that this difference was observed for each emotion (angry: *z* = 2.99, *P* = 0.003; happy: *z* = 2.60, *p*= 0.009; neutral: *z* = 2.81, *p* = 0.005). Moreover, as illustrated in Fig. 2B, the control group exhibited significant differences in their interpersonal distances according to the emotion presented. In particular, they preferred larger interpersonal distances when angry faces were approaching compared to happy and neutral faces (angry–neutral: *z* = 3.49, *p* = 0.0007; angry–happy: *z* = 6.059, *P* < 0.0001), in line with previous findings. By contrast, the patients displayed no significant difference in interpersonal distances depending on the emotion presented (all |*z*| < 0.5, *p* = 0.89).

In the real setting, the mixed-effect GLM revealed a significant main effect of Group [*F*(1,11.16) = 10.68, *p* = 0.007] and Condition [active–passive: *F*(1,224.04) = 20.24, *p* < 0.001]. No significant interaction between Group and Condition was found [*F*(1,224,04) = 0.82, *p* = 0.37]. As with the Bayesian test of deficit (Crawford and Garthwaite 2007), post-hoc analysis revealed larger interpersonal distances for control subjects compared to patients (controls–patients: *z* = 3.27, *p* = 0.001). The analysis also showed larger interpersonal distances in the passive condition compared to the active condition but only in the control group despite the lack of significant interaction between Group and Condition (active–passive: controls *z* = −4.48 *p* < 0.0001; patients *z* = −1.01 *p* = 0.31).

### Heart rate modulation with looming faces stimuli in the virtual reality setting (experiment 1)

For each participant, we computed the difference in mean HR frequency during the presentation of looming faces and the mean HR frequency during the baseline period (fixation cross) (i.e. delta HR; see methods and Fig. 2C). The statistical comparison based on the Bayesian test of deficit (Crawford et al. 2011) of the delta HR between each patient (P1 to P3) and the control group is presented in Table 2. This analysis only shows a significant difference between P1 and the control group [M ± SD = −0.58 bpm ± 0.64, P1 = 0.77, Zcc = 2.12, PA = 3.99, *p* = 0.04; P2 = 0.37, PA = 9.63, Zcc = 1.49, *p* = 0.09; P3 = 0.33, PA = 10.73, Zcc = 1.42, *p* = 0.11], with a lower delta HR for control group compared to P1 in response to looming faces.

The mixed-effect GLMs analysis revealed a significant effect of Group (*F*(1, 13.64) = 6.36, *p* = 0.02) with post-hoc tests indicating that the control group had significant lower delta HR frequency compared to patients (controls–patients: *z* = −2.51, *p* = 0.01). However, no significant effect of Emotion (*F*(2, 1197.9) = 1.43, *p* = 0.24) nor any significant interaction between Group and Emotion (*F*(2, 1198.06) = 1.38, *p* = 0.25) was found. Details of HR values obtained for each emotion are presented in the Supplementary Materials (Table S1).

### Evaluation of emotional rating: VT

The results show that all 3 patients and the control group correctly categorized happy, neutral, and angry emotions. As reported in Table 3, the rating of emotions did not statistically differ between patients and controls, demonstrating that the patients were not impaired in recognizing facial emotions (all *p* values > 0.05).

**Table 3:** Comparison of emotional rating of face stimuli in the VT in patients (P1, P2, P3) and controls (*n* = 10), assessed with the Bayesian test of deficit (Crawford and Garthwaite 2007).

	Angry			Neutral			Happy		
	Patient’s score	Controls score	*P* ^*^	Patient’s score	Controls score	*P* ^*^	Patient’s score	Controls score	*P* ^*^
P1	−2.55	−2.12 ± 0.48	0.21	−0.25	−0.31 ± 0.69	0.53	3.05	2.44 ± 0.47	0.88
P2	−2.65		0.16	−0.10		0.61	2.55		0.59
P3	−2.20		0.44	−0.95		0.2	2.55		0.59

^*^One-tailed *T*-tests.

## Discussion

This study presents evidence of a dysfunctional regulation of interpersonal distances following unilateral (left- or right-sided) medial–temporal lesions, including or not the amygdala. Compared to neurotypical control individuals, patients with left (P1 and P2) or right (P3) medial–temporal lesions display shorter interpersonal distances on two versions of the stop distance paradigm, in VR (experiment 1) and in real (experiment 2) conditions. The lesion extent varied across patients and included a large portion of the amygdala in P1, whereas in P2 and P3, the lesion spared most or all of the amygdala, respectively, affecting other regions of the temporal lobe. This indicates that the regulation of interpersonal distances does not depend only upon the integrity of both amygdalae; rather, a unilateral lesion within the temporal lobe might be sufficient to affect this physical regulation of distances that govern social interactions.

The actual physical distances preferred by control participants in the virtual and real settings are in line with preferred distances previously reported in the literature (Iachini et al. 2014, 2015, 2016; Ruggiero et al. 2017). Note that interpersonal distances measured in the VR setting were larger than those measured in real settings, an effect that might be related to differences in perceptual judgment from an egocentric perspective in both settings (Renner et al. 2013; Feldstein 2019). As also previously reported (Ruggiero et al. 2017; Cartaud et al. 2018), we found significant differences in interpersonal distances depending on facial emotion in controls, with larger interpersonal distances for angry faces compared to happy and neutral faces. This larger distance can be viewed as an avoidance response to the violation of personal space, an important biological adaptive mechanism to ensure the survival of the organism (Darwin 1872; Hediger 1955; Adams et al. 2006; Van Dantzig et al. 2008). It is generally admitted that emotional signals during social interactions allow us to anticipate others’ intentions by regulating spatial distances around us (Knutson 1996; Ruggiero et al. 2017).

Here, we report that this adaptive behavior is abolished following unilateral damage to the temporal lobe. Indeed, all three patients consistently displayed significantly shorter interpersonal distances compared to the control group in both settings, real and virtual. Interestingly, the interpersonal distances for the three patients were in a similar range to that of the single case patient (SM) with a selective bilateral damage to the amygdala (Kennedy et al. 2009). Also, similar to that study, P1 and P2 explicitly reported not feeling uncomfortable with the proximity of the real experimenter or the looming faces in the immersive VR environment. Yet, contrary to SM, the three patients selected in the present study had unilateral lesions that, in addition, were not restricted to the amygdala. This finding indicates that a unilateral medial–temporal lesion might be sufficient to disrupt the regulation of social distances and, notably, this holds true regardless of the side of the hemispheric damaged. The second novel result of this study is that, contrary to control subjects, none of the patients modulated interpersonal distance according to the emotion displayed by the approaching faces. Noteworthy, all three patients correctly categorized the facial emotional expressions, ruling out the possibility that the lack of modulation by facial emotional expression reflected a deficit in emotion recognition. This finding suggests, instead, that unilateral damage to the temporal brain regions, not limited to the amygdala, impairs the ability to adjust appropriately physical distance in social contexts. Finally, in the virtual setting (exp 1), we also measured the HR frequency of controls and patients while the emotional faces were looming toward them. We found a significant modulation in HR compared to baseline in controls, while no changes were detectable in patients. The results in controls resonate with previous evidence reporting an increase of HR frequency (Dureux et al. 2021) or skin conductance response (Candini et al. 2021) associated with social proximity.

However, in the present study, we found an opposite trend, that is a decrease rather than an increase of HR with looming faces. The apparently contrasting effect could be due to methodological differences as previous studies have typically compared physiological responses in different static, spatial positions (close and far from the participants) whereas in the present study, we did not use static stimuli, but we compared HR responses during the entire looming phase where the faces approached from far to close distances (i.e. period of 6 s) with the fixation cross period, preceding the approaching face. Our results also showed no significant effect of the emotion depicted by faces in either patients or control group which, for the control group, is at odds with previous studies that reported modulation of skin conductance response and cardiac inter-beat interval with emotional faces (Cartaud et al. 2018; Ruggiero et al. 2021). In a previous study, we found no difference in HR responses but only an effect of emotion on heart-rate variability (HRV) responses according to the emotion depicted by faces (Dureux et al. 2021). Therefore, future studies might help better understand these differences, for example with the use of different physiological measures and/or the use of different static spatial positions.

Importantly to the current context, the HR response profile to approaching faces differed between patients and controls, suggesting that only in the control group, these stimuli elicited HR changes while these changes were absent or not detectable in the patients. These differences between controls and patients might be related to the impact of the medial temporal lobe lesion onto the regulation of autonomic responses (Gloor et al. 1982; Inman et al. 2020).

Within the medial temporal cortex, the amygdala plays a key role in understanding social situations (Martin and Weisberg 2003; Noack et al. 2015) and in detecting social violations (Bas-Hoogendam et al. 2017). This is coherent with the inappropriate regulation of social distance in patient SM with bilateral amygdala damage (Kennedy et al. 2009). Focusing on the extent of amygdala lesions in each patient included in the present study, we note that P1 has a large part of his left amygdala affected (64% of lesioned voxels), while in P3 the right amygdala was largely spared by the lesion (1% of lesioned voxels) and in P2 the amygdala was intact. In addition, the time interval between surgery and testing differed between patients (1–60 months), the longer time interval being for P1. This difference, together with the lesion location and extent could explain why P1 stands out from the other 2 patients with for instance the shortest preferred interpersonal distance in the real setting and the highest delta HR compared to the other 2 patients P2 and P3.

Yet, our results further indicate that other regions of the temporal lobe, including the parahippocampal cortex, the hippocampus, the fusiform gyrus, the temporal pole, and the middle temporal gyrus, might participate in the regulation of interpersonal distances. Among these regions, the temporal pole, that has received less attention than the amygdala, has also been involved in socio-emotional processing and facial recognition in both monkeys and humans, as well as in higher-level social cognition functions such as empathy and theory of mind essential for interpersonal relationships (for a review, see Herlin et al. 2021). Another, not mutually exclusive, possibility is that the resection of temporal lobe tissue in the 3 patients, and perhaps more so in P2 and P3, may have led to a disconnection of the amygdala and/or the temporal pole, through damage to white matter tracts ([Bibr ref57][Bibr ref57], 2020). Recent studies have shown that functional disconnection of white matter tracts and in particular of the superior longitudinal fasciculus (SLF/AF), connecting the frontal, occipital, parietal, and temporal lobes (Schmahmann et al. 2008), impairs mentalizing processes (Herbet et al. 2015; Yordanova et al. 2017), emotional processes (Herbet et al. 2018), and other personality disorder as well as risky behaviors (Jiang et al. 2017; Herbet et al. 2018). The resection of temporal lobe tissue may have also damaged other bundles of fibers among which the uncinate fasciculus, a major tract connecting the temporal lobe with medial frontal cortex (Kier et al. 2004). A study has shown, for instance, that a disconnection of the left uncinate fasciculus is associated with positive schizotypal traits (Lemaitre et al. 2018), while damage to the right uncinate fasciculus is associated with impairment in emotional empathy (Oishi et al. 2015). It would therefore be interesting to investigate in future studies the integrity of these white matter fiber tracts in patients undergoing resection of temporal lobe tissue together with their ability to regulate interpersonal distances. Importantly though, the three patients included in the present study underwent a complete neuropsychological assessment and none of them showed any sign of cognitive deficit (see Methods, only P3 who had a mild impairment in long-term verbal memory). Finally, the psychological scale administered to the patients’ caregiver (i.e. wife) revealed no difference for any of them in their ability to keep and maintain social rules.

In conclusion, our behavioral and physiological results provide the first evidence that a unilateral damage to the medial–temporal lobe, including or not the amygdala, appears to be sufficient to induce inappropriate regulation of social distances, despite the preserved ability of the patients to maintain social rules in their daily life. In keeping with previous reports, our findings support the view that social interpersonal behaviors, with their underlying mechanisms (among which the regulation of interpersonal space), rely on interconnected networks that depend on the integrity of both medial temporal cortex.

## Supplementary Material

Supplementary_materials_CCC-2022-00018_R1_tgac031
