# Supplementary material for: Personal space regulation is affected by unilateral temporal lesions beyond the amygdala
Source: Cereb Cortex Commun. 2022 Jul 22;3(3):tgac031. doi: 10.1093/texcom/tgac031 (PMC9441012; doi:10.1093/texcom/tgac031)
Supplement: Supplementary_materials_CCC-2022-00018_R1_tgac031 [file supplementary_materials_ccc-2022-00018_r1_tgac031.pdf]

# Supplementary materials

**Table S1. Details of HR responses as a function of the emotion depicted by faces for patients (P1, P2, P3) and controls (n=10) (i.e., mean HR, mean baseline and mean delta HR (i.e., mean HR for looming faces – mean HR during fixation cross))**

| | Patients | | | | Control group | | | |
|---|---|---|---|---|---|---|---|---|
| **Emotion depicted by faces** | | **Mean HR** | **Mean Baseline** | **Delta HR** | **n** | **Mean HR** | **Mean Baseline** | **Delta HR** |
| *Happy* | **P1** | 79.21 | 78.25 | 0.96 | 9 | 71.68 | 71.96 | -0.28 |
| | **P2** | 65.31 | 64.40 | 0.91 | | | | |
| | **P3** | 101.07 | 104.26 | -3.18 | | | | |
| *Neutral* | **P1** | 80.48 | 79.03 | 1.45 | 9 | 71.26 | 71.71 | -0.45 |
| | **P2** | 66.34 | 65.76 | 0.58 | | | | |
| | **P3** | 105.38 | 101.61 | 3.77 | | | | |
| *Angry* | **P1** | 79.35 | 79.45 | -0.10 | 9 | 71.61 | 71.96 | -0.35 |
| | **P2** | 65.42 | 65.82 | -0.40 | | | | |
| | **P3** | 105.89 | 105.30 | 0.59 | | | | |
